# Ovarian Cancer: Can Proteomics Give New Insights for Therapy and Diagnosis?

**DOI:** 10.3390/ijms14048271

**Published:** 2013-04-15

**Authors:** Angela Toss, Elisabetta De Matteis, Elena Rossi, Lara Della Casa, Anna Iannone, Massimo Federico, Laura Cortesi

**Affiliations:** 1 Department of Oncology & Haematology, University of Modena and Reggio Emilia, Modena 41125, Italy; E-Mails: angela.toss@tin.it (A.T.); elisabetta.dematteis@unimore.it (E.D.M.); mfederico@unimore.it (M.F.); 2 ProteoWork Lab, the Department of Biomedical Sciences, University of Modena and Reggio Emilia, Modena 41125, Italy; E-Mails: elena.rossi@unimore.it (E.R.); lara.dellacasa@unimore.it (L.D.C.); anna.iannone@unimore.it (A.I.)

**Keywords:** proteomic, ovarian cancer, biomarker, therapy, OVA1

## Abstract

The study of the ovarian proteomic profile represents a new frontier in ovarian cancer research, since this approach is able to enlighten the wide variety of post-translational events (such as glycosylation and phosphorylation). Due to the possibility of analyzing thousands of proteins, which could be simultaneously altered, comparative proteomics represent a promising model of possible biomarker discovery for ovarian cancer detection and monitoring. Moreover, defining signaling pathways in ovarian cancer cells through proteomic analysis offers the opportunity to design novel drugs and to optimize the use of molecularly targeted agents against crucial and biologically active pathways. Proteomic techniques provide more information about different histological types of ovarian cancer, cell growth and progression, genes related to tumor microenvironment and specific molecular targets predictive of response to chemotherapy than sequencing or microarrays. Estimates of specificity with proteomics are less consistent, but suggest a new role for combinations of biomarkers in early ovarian cancer diagnosis, such as the OVA1 test. Finally, the definition of the proteomic profiles in ovarian cancer would be accurate and effective in identifying which pathways are differentially altered, defining the most effective therapeutic regimen and eventually improving health outcomes.

## 1. Introduction

Ovarian cancer represents one of the four main gynecological cancers, counting worldwide about 225,000 new cancer cases diagnosed in 2008 (3.7% of all female cancers) and about 140,000 deaths (4.2% of all deaths in women) [1]. According to the American Cancer Society, in 2012, ovarian cancer was expected to account for 3% (22,280) of all new cases and 6% (15,500) of all female cancer deaths in the United States. The proportion of ovarian cancer among gynecological cancers is increasing; on the other hand, survival for ovarian cancer is the poorest of all gynecological cancer sites, with a five-year relative survival rates of 44% for all stages [2]. The main reasons for this poor survival are the lack of early detection strategies and an unfavorable anatomical situation. Thus, the vast majority of ovarian cancer is diagnosed at an advanced stage, and therapy for this pathology is very complex. Reduction in mortality rates could be gained from progresses in surgery and medical treatments and finding the correct role of an organized screening program. A major impact on the mortality from ovarian cancer and increases in survival rates could be gained translating recent insights at the molecular and cellular level to improve early detection and to personalize treatment strategies.

Proteomic technology has emerged in the last decade as a powerful tool to explain the molecular mechanisms of malignant cells and tumor microenvironment in patients with cancer. Proteomics has given useful insight into the perturbations of signaling pathways within tumor cells, allowing one to find new targets for drug action, possible diagnostic markers and prognostic indicators of outcome and response to therapy [3,4]. The proteome represents all the possible gene products of a cell. Any protein may exist in multiple forms that vary within a particular cell or in different cells, because of modifications derived from translational, post-translational, regulatory and degradative processes that affect protein structure, localization, function and turnover. Proteomic techniques characterize all proteins in a biological system, including complicated features, like isoforms, modifications, interactions and functional structures. Particularly in cancer biomarker discovery, two-dimensional gel electrophoresis, mass spectrometry (MS) and protein microarrays, in conjunction with advanced bioinformatics, have become useful devices to identify proteins.

This review summarizes the most frequent genetic pathways of sporadic and hereditary ovarian cancer, focusing on proteomic strategies aimed at identifying early diagnostic biomarkers, prognostic indicators of outcome and response to therapy and possible targets for drug action.

## 2. Genetic Pathways of Sporadic Ovarian Cancer

Ovarian cancer represents a heterogeneous disorder with different biology and behavior at the clinical, cellular and molecular levels. The normal ovary tissue is characterized by several distinct components. About 10% of ovarian cancers develop from germ cells or granulosa-theca cells, while more than 90% of ovarian cancers has an epithelial origin and arises from cells that cover the ovary. However, cancers with similar histology can also arise from the lining of the fallopian tube, deposits of endometriosis or the surface of the peritoneal cavity [5]. Epithelial ovarian cancer is divided into four main histotypes, with a differentiation similar to the normal cells that line the fallopian tube (serous), endometrium (endometrioid), endocervix (mucinous) and vagina (clear cell). At a molecular level, the gene expression profiles of different ovarian cancer histotypes correlate with their morphological counterparts in normal tissues [6]. Particularly, histotypes correlate with the abnormal re-expression of homeobox (Hox) genes that are normally only expressed during gynecological organogenesis. Respectively, HOXA9 is expressed in the fallopian tube and serous ovarian cancer, HOXA10 in uterus and endometrioid cancer and HOXA11 in lower uterine segment and cervix and mucinous cancers [7]. On this basis, the classic theory regarding the role of the ovarian surface epithelium in ovarian cancer pathogenesis has been recently reconsidered. A novel hypothesis supports the direct involvement of the distal fallopian tube in the development of serous carcinomas and ectopic endometrium in endometrioid and clear cell carcinomas [8].

**Figure 1:**
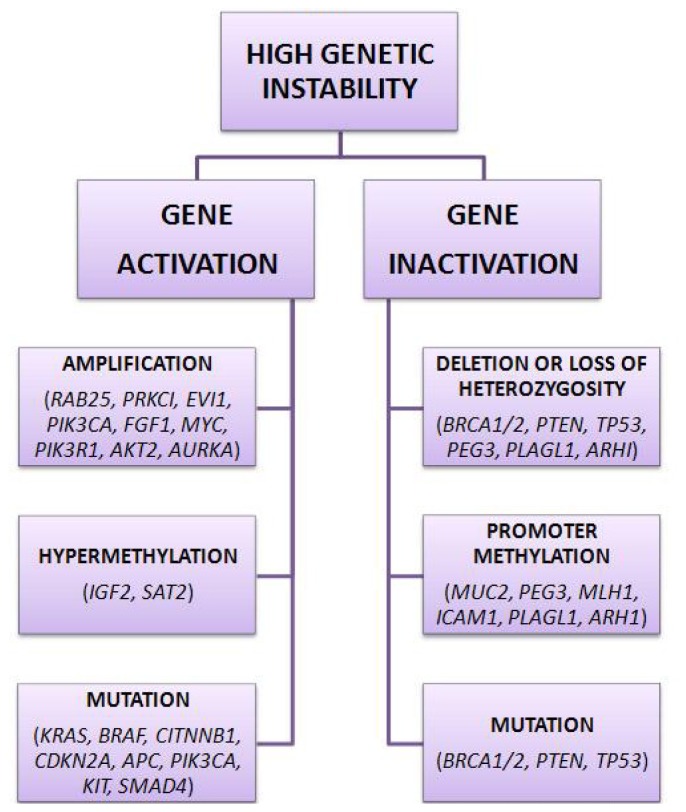
Several genetic alterations cause of high genetic instability in ovarian cancer.

Protein expression analysis has been widely introduced in order to identify a signature that may characterize each distinct histotype. Apart from pathogenesis of different histotypes, the common genetic pathway of sporadic ovarian cancer is based on high genetic instability as a result of the activation or inactivation of specific genes. Activation of genes occurs through amplification, mutation and hypomethylation, whereas genetic inactivation results from deletion of large chromosomal regions, loss of heterozygosity at specific loci, mutations and promoter methylation (Figure 1).

Within this wide genetic panel, a total of 16 tumor suppressor genes, a total of 15 oncogenes and three imprinted tumor suppressor genes can be identified (Table 1) [5,9]. Notably, the function of imprinted tumor suppressor genes can be lost in a single step, since only one allele is expressed in each cell, due to the inheritance of a silent maternal or paternal allele. Of the 16 candidate tumor suppressor genes identified to date, three are imprinted genes.

On the basis of the genetic profile, ovarian cancer can be divided in two different groups. Type I ovarian cancers include low-grade and borderline serous cancers, endometrioid, mucinous and clear-cell cancers. This group has more frequent *PTEN*, PI3K catalytic subunit-α (*PIK3CA*), *KRAS*, *BRAF* and b-catenin (*CTNNB1*) mutations, along with genomic stability and *TP53* mutations in only a small fraction of cases. Type II ovarian cancers include high-grade serous carcinomas, mixed malignant mesodermal tumors, carcinosarcomas and undifferentiated cancers. These tumors have *TP53* mutations in up to 80% of cases, along with marked genomic instability and arise more frequently from the fallopian tubes and the peritoneum and in *BRCA1* and *BRCA2* mutation carriers [10]. Interestingly, mutated *TP53* has been observed in microscopic ovarian cancers found in oophorectomy specimens resected prophylactically from *BRCA1* or *BRCA2* mutation carriers, suggesting an early role of *TP53* in hereditary disease. Alteration of *TP53* function is one of the most frequent genetic abnormalities in ovarian cancer and is observed in 60%–80% of both sporadic and familial tumors. Overexpression of this tumor suppressor gene is seen in approximately 4% of preinvasive borderline tumors, 10%–20% of early cancers and 40%–60% of advanced cancers and correlates with metastatic potential. Moreover, *TP53* mutation has been associated with resistance to platinum-based therapy in some studies and a short-term survival benefit [11].

**Figure 2:**
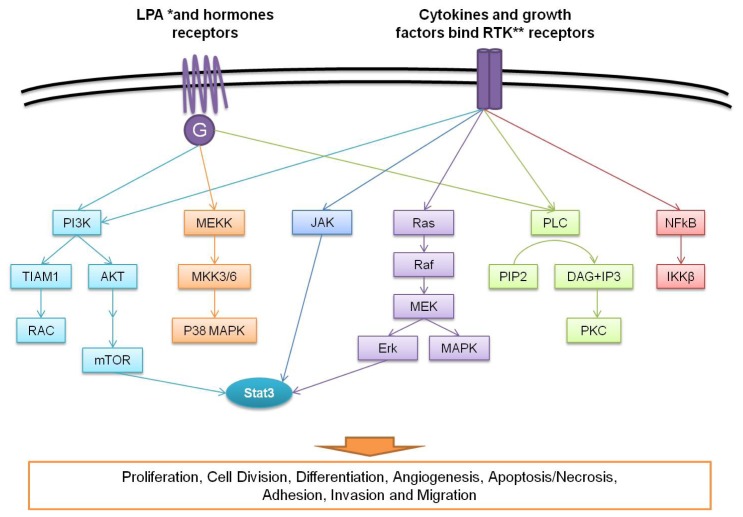
Signaling pathways in ovarian cancer pathogenesis. * Lysophosphatidic acid; ** Receptor tyrosine kinases.

In ovarian cancer pathogenesis, the sequence of events includes the activation of several signaling pathways, which consist of several molecules intervening in cancer cell metabolism, proliferation, differentiation, movement and apoptosis. According to the literature, the signaling pathways involved in ovarian cancer pathogenesis are the following: the lysophosphatidic acid (LPA) pathway involved in 90% of cases, the phosphatidylinositol 3-kinases (PI3K) pathway involved in 70% of cases, the nuclear factor kappa-light-chain-enhancer of activated B cells (NF-kB) pathway involved in more than 50% of cases, the mitogen-activated protein kinase (MAPK) pathway involved in less than 50% of cases, the proto-oncogene tyrosine-protein kinase Src pathway, the Müllerian Inhibitory substance receptor pathway, the ErbB activation pathway involved in approximately 25% of cases, the epidermal growth factor (EGF) and vascular endothelial growth factor (VEGF) pathways, the endoplasmic reticulum (ER) beta pathways, the activator of transcription 3 (Jak-STAT 3) pathway, the Interleukin-6/Interleukin-6 receptor (IL-6/IL-6R) or the Janus Kinase 2 signal transducer pathways (Figure 2) [5,12]. The knowledge of a proteomic signature in ovarian cancer permits one to develop inhibitors of the molecules that are most frequently activated or overexpressed in ovarian cancers.

## 3*. BRCA*-Related Ovarian Cancer

The most important risk factor for ovarian cancer still remains a family history of breast or ovarian cancer. Up to 10% of ovarian cancer patients may have inherited a germline mutation that places them at increased risk for the disease. Mutations in the breast and ovarian cancer-susceptibility genes, *BRCA1* and *BRCA2*, confer an increased lifetime risk of ovarian cancer. However, *BRCA* mutations do not account for the entire range of hereditary ovarian cancer syndromes. Other hereditary epithelial ovarian cancers are attributed to Lynch syndrome. Lynch syndrome is an autosomal dominant disorder, which predisposes to colorectal, endometrial, ovarian, gastric, small bowel, biliary/pancreatic, urothelial, skin and central nervous system cancers. The cumulative risk of ovarian cancer is estimated to be 8%–10%, with an average age of onset of 42 years [13]. Moreover, other genes often associated with rare cancer syndromes, such as *TP53* and *PTEN* or *CHEK2* and *PALB2*, confer a low to moderate risk for breast and ovarian cancer [14,15]. Recent technological advances have aided in the recognition of additional tumor suppressor genes potentially associated with hereditary breast and gynecologic cancer, such as *RAD51* and *BARD1*[16]. To date, at least 16 genes have been associated with hereditary ovarian cancer, mostly involved in the *FA-BRCA* pathway and the mismatch repair system. However, many families with suspicious pedigrees do not have a specific mutation identified through clinical testing, due to a currently undetectable *BRCA1/2* mutation or a mutation in another susceptibility gene.

Inheritance of DNA repair defects contributes to as many as 10%–15% of ovarian cancers. About 90% of hereditary ovarian cancer derives from germline mutations in *BRCA 1/2* genes. Women who have inherited genetic mutations have substantially increased risk of ovarian cancer, with a lifetime risk that varies with the genetic defect (for *BRCA1*, 30%–60%, for *BRCA2*, 15%–30% and for hereditary non-polyposis colon cancer, 7%) [17]. Particularly, BRCA-related ovarian cancers have characteristic behaviors. They are more frequently multifocal, with genetically distinct clones involving multiple sites and progress faster. These kinds of tumors are more sensitive to platinum-based chemotherapy and poly(ADP-ribose) polymerase (PARP) inhibitors, which are particularly effective in the presence of defects in homologous recombination repair. However, germline mutation in *BRCA1/2* genes can be involved even in the pathogenesis of BRCA1-like and BRCA2-like sporadic ovarian cancer [9].

## 4. Proteomic Techniques

### 4.1. Two-Dimensional Gel Electrophoresis (2DE)

Traditionally, proteomic analyses have been performed using two-dimensional gel electrophoresis (2DE), which separates proteins according to two distinct protein characteristics, size and charge. The introduction of immobilized pH gradients and advanced bioinformatic approaches have recently improved the reproducibility and comparability of this technique [18]. In several studies, 2DE has been able to detect differences between normal and cancer sample proteomes [19].

The analysis of the proteome started out with techniques based on 2DE and then extended with liquid chromatography–mass spectrometry (LC–MS)-based techniques. However, despite the rapid advance of MS-based proteomics, 2DE still plays a crucial role in several fields of protein and molecular biology. There is an important niche for 2D gel-based proteomics, which complete traditional LC-MS techniques, in the areas of protein identification from organisms with no or incomplete genome sequences available, alternative detection methods for modification of specific proteomics and identification of protein isoforms and modified proteins [20].

### 4.2. Mass Spectrometry (MS)

Mass spectrometry (MS) provides a snapshot of a proteome in time and space, determining the precise mass and charge (*m/z*) of proteins and, thus, identifying the actual precursor proteins or protein profiles. In recent years, MS instruments have been greatly modified and improved, becoming sensitive to the picomole to femtomole range required for the detection of oligonucleotides, small polar molecules, peptides, proteins and post-translationally modified proteins, such as glycoproteins and phosphoprotein [21]. Mass spectrometers are composed of an ion source, a mass analyzer and an ion detector. Different combinations of ionization sources (such as MALDI and ESI), mass analyzers (such as time-of-flight (TOF), quadrupole and ion traps) and fragmentation methods (such as CID (collision-induced dissociation), ETD (electron-transfer dissociation) and ECD (electron-capture dissociation)) can be used.

Although 2DE is still used in several proteome studies, the shotgun mass spectrometry (shotgun-MS) has been widely introduced in recent years. A shotgun-MS strategy consists in the separation of peptides produced from protein digestion in complex mixtures followed by tandem mass spectrometry (MS/MS) analysis. Traditional shotgun proteomics is a useful tool for the analysis and identification of proteins and their variations in biological samples with a non-targeted approach.

Biological samples are often heterogeneous and contain several different molecules; thus, methods of separation are often necessary to select the target to analyze from the other molecules in the sample. Gas (GC) or liquid chromatography (LC) are common methods of pre-MS separation used when analyzing complex gas or liquid samples. High performance liquid chromatography (HPLC) is the most common separation method to study biological samples by MS or MS/MS (termed LC-MS or LC-MS/MS, respectively). Traditionally, the first step is sample lysis and protein extraction from the cells, tissues or body fluids, followed by digestion into peptides. This process generates thousands of peptides, which can be pre-fractionated according to their physicochemical properties (such as charge or isoelectric point) or enriched by different means (for example, affinity resins or specific antibodies). These pre-fractionated or enriched samples are then analyzed one-by-one by reversed-phase liquid chromatography coupled to mass spectrometry (LC–MS), in which selected peptides are fragmented by tandem mass spectrometry (MS/MS). MS and MS/MS are connected to computer software that analyzes the detected ions by their individual *m*/*z* and relative abundance. These ions can then be processed through databases to identify the corresponding peptide sequences [22,23].

Among the MS-based proteomics approaches, matrix assisted laser desorption/ionization mass spectrometry (MALDI MS) and surface enhanced laser desorption/ionization mass spectrometry (SELDI MS) have been employed for large scale polypeptide profiling in clinical proteomics [19]. Proteomic profiling with MALDI and SELDI MS has been used to investigate the spatio-chemical make-up of a tissue, identifying peptides and proteins that are specific to the different cell types that characterize a tumor. Particularly, these techniques have been applied to identify new candidate ovarian cancer biomarkers [24].

MS-based proteomics is a universally used technique for analyzing complex protein samples. To visualize the spatial distribution of specific molecules in biological systems, an increasing number of groups are looking to combine the sensitivity and specificity of mass spectrometry with imaging capabilities. This involved both of the macromolecular ionization techniques, MALDI and ESI and provided a foundation for secondary ion mass spectrometry (SIMS) imaging. Since MALDI provides proteomics information and SIMS that of lipids and other surface active species, several groups now use the complementary information provided by SIMS and MALDI to obtain proteomics, lipid and vitamin distributions within the same tissue section [25]. However, MALDI-mass spectrometry Imaging (MALDI-MSI) has not yet been used to map the transcriptome, which includes microRNA and other RNA-related molecules, and represents now a wide area of clinical research. To investigate this important topic, targeted mass spectrometric imaging (Tag-Mass MSI) has been developed. Particularly, targeted MSI may be used to complement MALDI-MSI for clinical research. For instance, using a candidate ovarian cancer biomarker (the *C*-terminal fragment of the activator of the immunoproteasome, PA28 alpha), it is possible to detect the protein and the mRNA encoding this protein in the same sample. Since the result showed that the level of transcription is related to the abundance of the protein fragment, cross-validation between the transcriptome and the proteome can be performed on the same tissue. To conclude, targeted MSI associated with classical immunohistochemistry (IHC) may offer the possibility to obtain a quick answer and a better molecular diagnosis for clinical surgery [26].

These technologies enabled the detection of low-abundance proteins, identifying hundreds of potential biomarkers with diagnostic, prognostic and therapeutic value. However, there is a lack of analytical validation of platforms for the precise measurements of the candidate markers in a smaller set of samples before lengthy and costly large-scale clinical trials. In this context, targeted proteomic technologies, such as multiplex MS, protein arrays and several variations of enzyme-linked immunosorbent assays (ELISAs) have provided a sensitive and specific quantitative measurement of protein targets in biological systems. The accuracy and statistical rigor of these technologies may be used to investigate the molecular mechanism of cellular function through the analysis of protein networks and pathways and to confirm and triage hundreds of potential biomarkers prior to large-scale clinical trials. Stable isotope dilution (SID) multiple reaction monitoring MS (SID-MRMMS) has emerged as one of the powerful targeted proteomic tools in the past few years [27].

### 4.3. Protein Microarrays

Malignant transformation of ovarian epithelial cells is due to genetic mutations that compromise regulation of proliferation, differentiation and apoptosis. Since a close connection exists between genetic alterations and tumorigenesis, research on gene level (including inherited gene mutations, epigenetic changes and gene expression) could provide important biological, diagnostic and prognostic information. DNA or RNA-based cancer biomarkers utilize microarrays, polymerase chain reaction (PCR), reverse transcriptase polymerase chain reaction (RT-PCR), DNA sequencing, fluorescent *in situ* hybridization (FISH), *etc.*, to detect the genetic alterations occurring in the cancerous state. In the last decade, microarray technology has allowed for the measurement of the expression of tens of thousands of genes in a given tissue sample and to compare gene expression between normal and malignant cells, identifying genes that are differentially regulated during cancer development [28].

Protein microarrays, similar to gene arrays, emerged as a promising technique to analyze the abundance of proteins and their alterations [19]. Protein microarrays are now being used to profile the proteome of cell populations using antigen-antibody interactions [29]. Protein microarray formats fall into two principal classes: forward phase arrays (FPA) and reverse phase arrays (RPAs). In forward-phase arrays (FPAs), antibodies are arrayed and probed with cell lysates, while in reverse-phase arrays (RPAs), cell lysates are arrayed and probed with antibodies [18, 30]. Contrary to FPAs, RPAs do not require labelling of cellular protein lysates and constitute a useful platform for biomarker screening, pathophysiologic studies and therapeutic monitoring. Furthermore, the RPA has a unique ability to analyze signaling pathways using small numbers of cultured cells or cells isolated by laser capture microdissection (LCM) from human tissues procured during clinical trials [30]. The major limitation for RPAs is the specific antibodies that are required. In ovarian cancer, the RPA platform has been used to study disease progression and profile signaling pathways, identify therapeutic targets and suggest prognostic indicators.

## 5. Proteomics and Post-Translational Modifications

Proteomic strategies are able to provide more detailed information about biological systems than the determination of the static genome or expression profiling based on mRNA. In contrast to the genome, the complexity of the proteome is increased by alternative splicing of mRNAs and a wide variety of post-translational events (such as glycosylation, phosphorylation and ubiquitination). For all these reasons, genomics and mRNA analysis do not correlate with protein content, while proteomics may study the presence of a specific protein and provide a measure of the quantity present.

Glycosylation is one of the most common post-translational modifications. The mechanism of glycosylation is involved in several biological processes, including cell communication, adhesion and signaling. Moreover, the association between aberrant glycosylation and disease development has promoted a number of research activities directed to provide reliable diagnostic or prognostic information [31]. MS glycomics methods have been successfully employed to compare the glycomic profiles of different samples collected from healthy individuals and ovarian cancer patients [32]. Particularly, Abbott *et al.* studied the differences in the transcripts of a restricted set of glycosyltransferases involved in N-linked glycosylation in ovarian cancer tissues and normal ovarian tissues, identifying candidate biomarkers with cancer-specific glycosylation, such as periostin and thrombospondin [33]. More recently, Shetty *et al.* found that several proteins are aberrantly sialylated in N-linked glycopeptides in ovarian cancer tissue, and detection of glycopeptides with abnormal sialylation changes may be exploited as biomarkers for ovarian cancer [34]. Finally, Kuzmanov *et al.* identified the sialome (*i.e.*, sialic acid included in glycoproteins) utilizing tandem mass spectrometry as a potential pool of novel biomarker candidates. In all of the samples analyzed, 579 glycosylation sites on 333 proteins were identified [35]. To conclude, the proteins identified through glycomics strategies could form the basis for future studies examining and quantifying their glycosylation status as biomarkers of ovarian cancer.

Reversible protein phosphorylation represents one of the most dynamic post-translational modifications. Rapid changes of protein phosphorylation largely influence signaling networks, playing a crucial role in the regulation of many cellular processes, including metabolism, differentiation, migration, proliferation and apoptosis. Phosphoproteomic strategies represent the optimal choice for studying phosphorylation-based signaling networks. The proteomics development in the area of MS has been improving with the breakthrough of quantitative MS through ICAT (isotope-coded affinity tag), iTRAQ (Isobaric tags for relative and absolute quantitation), isobaric peptide termini labeling (IPTL) and SILAC (stable isotope labeling by amino acids in cell culture). SILAC, the most extensively used method during the past years, represents an accurate and inexpensive procedure that can be used as a quantitative proteomic approach in any cell culture system [36]. Phosphoproteins co-exist with their unphosphorylated isoform within the cell and often are present in low quantity. Thus, to improve the identification of phosphoprotein, removing non-phosphorylated proteins from the sample and enriching for the phosphorylated isoforms is necessary before MS. This task is carried out by enriching techniques, such as immunoprecipitation, immobilized metal affinity chromatography (IMAC), metal-oxide affinity chromatography (MOAC), Phos-Tag chromatography, polymer-based metal ion affinity capture (PolyMAC), hydroxyapatite chromatography, enrichment by chemical modification and phosphopeptide precipitation [37]. Particularly, phosphoproteomics has played a significant role in investigating molecular mechanisms that govern oncogenesis. Phosphoproteomic strategies have been used to identify targets of kinase inhibitors, providing useful information to study their off-target effects therapeutic options [38].

## 6. Proteomic Biomarkers for Ovarian Cancer

One of the main goals of proteomics is the identification of biomarkers for diseases from tissues and body fluids. Current diagnostic tools have had very limited success in early detection of ovarian cancer. The search for an ovarian cancer screening modality with improved specificity and sensitivity has led to the examination of serum biomarker patterns using new “omic” technologies [39–41]. Several studies have analyzed the proteomic profiles of ovarian tumor tissue, cell lines, urine, ascites fluid and blood samples from ovarian cancer patients (Table 2) [42–46].

**Figure 3:**
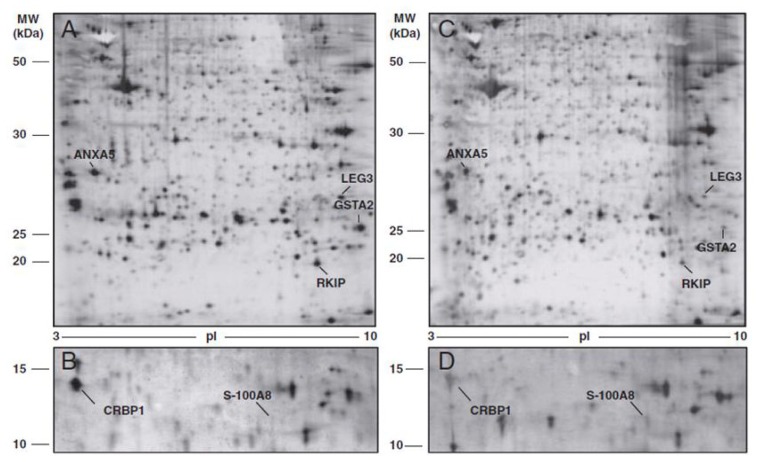
Comparison between proteome of normal ovarian tissue and the corresponding tumor tissue. Representative gels derived from normal ovarian tissue (**A** and **B**) and ovarian cancer tissue (**C** and **D**) of a single patient. A total of seven differentially expressed protein spots in tumor tissue are annotated and identified by mass spectrometry (MS) analysis.

**Figure 4:**
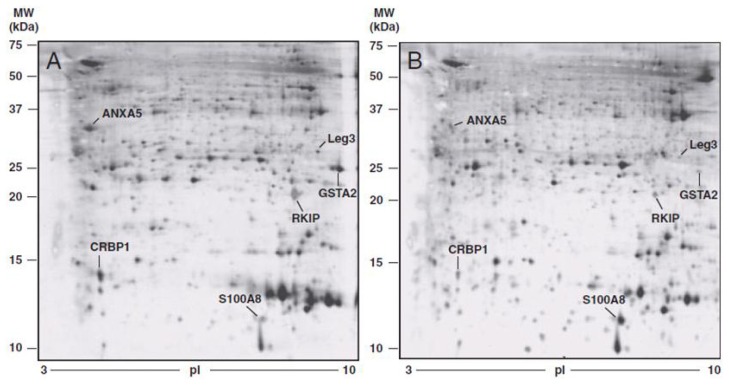
Comparison between proteins secreted by ovarian normal interstitial fluid (NIF) and by ovarian tumoral interstitial fluid (TIF). Two-dimensional gel electrophoresis (2DE) images of protein in NIF (panel **A**), obtained from healthy ovarian tissue biopsies and in TIF (panel **B**), obtained from ovarian cancer biopsies. Molecular weight (kDa) and isoelectric values (pI) are shown on the image. Arrows indicate proteins differentially expressed and identified by MS analysis.

A recent comparative proteomic study investigated and defined protein expression patterns associated with advanced stage ovarian cancer, to identify a panel of diagnostic or prognostic markers. The study also investigated proteins secreted by the cancer cell into the interstitial fluid, as cancer growth and progression also depends on stromal factors present in the tumor microenvironment. Moreover, many biomarkers present in biopsied cancer tissues can also be found in blood serum, representing potential biomarkers of the disease. Proteomic profiling of differentially expressed proteins in cancer ovarian tissue, tumoral interstitial fluid (TIF) and ascitic fluid, compared with healthy tissue sample and normal interstitial fluid (NIF), allowed for the identification of protein spots consistently differentially expressed between normal and cancer samples (Figures 3 and 4).

Protein expression/identification was evaluated by 2DE (two-dimensional gel electrophoresis) and MS (mass spectrometry) analysis and were confirmed by immunohistochemistry. Six proteins showed differential expression in tumoral interstitial fluid and tumor tissue compared to normal interstitial fluid and healthy tissue. Protein different expression between tumoral and normal ovarian tissue is presented in Table 3.

Five were found to be downregulated and identified as galectin 3, glutathione *S*-transferase A-2, retinol binding protein 1, phosphatidylethanolamine-binding protein and annexin 5, while the calgranulin was significantly upregulated in all pathological samples, including the ascitic fluid. This is the first study to report an overexpression of calgranulin by 2DE associated with MS/MS analysis on surgical biopsy. As previously reported, the reduced expression of galectin 3 and retinol binding protein 1 in cystic fluid and serum of patients with early stage disease is confirmed in this study. The results highlight alterations in proteins that control cell-cycle progression and apoptosis, as well as factors that modulate the activity of signal transduction pathways [42].

Petri *et al*. [43] examined whether urine could be used to measure specific ovarian cancer proteomic profiles and whether one peak alone or in combination with other peaks or CA125 had the sensitivity and specificity to discriminate between ovarian cancer pelvic mass and benign pelvic mass. Twenty-one significantly different peaks (*p* < 0.001) were visualized, and the three most significant peaks were identified as the fibrinogen alpha fragment, collagen alpha 1 (III) fragment and fibrinogen beta NT fragment. These results supported the feasibility of using urine as a diagnostic tool and suggested the enhanced prediction performance of combined marker analysis.

Li *et al.*[44] performed a comparative proteomic study of normal ovarian epithelial and ovarian epithelial serous cystadenocarcinoma tissue and identified six proteins significantly differentially expressed. Particularly, Prx-II expression was found linearly decreased from normal ovarian tissue, benign ovarian lesions and to ovarian malignancies. No statistically difference between carcinomas groups in different clinical stage, differentiation status and histological type was seen, suggesting that the decrease level of prx-II is a common marker for ovarian malignancies. This represented the first report on the altered expression of prx-II in ovarian cancer.

Jackson *et al.*[45] identified the vitamin E-binding plasma protein, Afamin, as a potential novel tumor marker, using proteomic-based approaches. This protein, found decreased in patients with ovarian cancer, was then analyzed in a large case-control study to evaluate the diagnostic utility. The study indicated that Afamin alone is not sufficiently suitable as a diagnostic marker, but can increase the Ca 125 sensitivity a little [47]. Recently, the prognostic value of Afamin, alone and in combination with Ca 125 has been evaluated in serum samples of patients with first diagnosis of primary ovarian cancer. Afamin, measured before surgery and after platinum-based chemotherapy, showed no correlation with any considered parameters, such as FIGO stage, residual post-operative tumor, platinum sensitivity, histological subtype, grade and age [48].

An *et al.*[46] identified that different histologic subtypes of ovarian malignant epithelial tumors showed distinctively different protein expression profiles. The potential candidate biomarkers screened in ovarian tumors found to be significantly upregulated in comparison to normal tissues were: protein phosphatase-1, ferritin light chain, proteasome R-6 and NAGK (*N*-acetylglucosamine kinase).

In 2008, the FDA approved human epididymis protein 4 (HE4) to monitor recurrence or progression of epithelial ovarian cancer. Moreover, reliable clinical evidence demonstrates that HE4, alone or in combination with CA125, improves the accuracy of screening [49]. HE4 is a secreted glycoprotein overexpressed by serous and endometrioid ovarian cancers and expressed by 32% of ovarian cancers with non-elevated CA125 [50]. The analysis of this protein represents a valuable tool for ovarian cancer diagnosis, but, when combined with CA125, the marker acquires a higher sensitivity, providing a more accurate predictor of ovarian cancer than alone [51]. Notably, Moore *et al.* published a series of papers that used a combination of CA125, HE4 and menopausal status to predict the presence of a malignant ovarian tumor and developed the Risk of Ovarian Malignancy Algorithm (ROMA), a simple biomarker-based algorithm, which requires ultrasound [52,53].

Since ovarian cancer represents a complex and heterogeneous disorder, it is unlikely that a single biomarker could be able to discriminate between healthy women and patients with ovarian cancer. The use of a combination of biomarker candidates could provide higher specificity and sensitivity for early detection of ovarian cancer [54,55]. On this basis, several novel multiplex assays that use combinations of different biomarkers have been developed. The combination of leptin, prolactin, osteopontin, insulin-like growth factor II, macrophage inhibitory factor and CA 125 yielded 95.3% of sensitivity and 99.4% of specificity, providing a potential useful alternative to screening with CA 125 alone for the diagnosis of ovarian cancer [56]. Different combinations of HE4, mesothelin, CEA, VCAM-1, B7-H4 and YKL-40 in association with CA 125 are under study for early detection. Other promising biomarkers include KLK6/7, GSTT1, prostasin (PRSS8), FOLR1 and ALDH1 [57]. Moreover, a combination of human cationic antimicrobial protein-18 (hCAP-18), Lactoferrin and CD163 has been analyzed in normal healthy women and ovarian cancer patients, finding a multivariate index assay with significantly increased diagnostic efficiency. The biomarker panel is worthy of further investigation in a larger phase 2 trial [58]. However, several questions about the current paradigm for biomarker development still remain open. If markers discovered in diagnostic samples are differentially expressed only when the tumor becomes large, it is possible that such markers may have little value for early detection. In this regard, Moore *et al.* evaluated several biomarkers alone and in combination with CA 125 in prediagnostically collected sera from women in the Prostate, Lung, Colorectal and Ovarian Cancer Screening trial. In contrast to the prior findings on post-diagnostically collected samples, the addition of these biomarkers to CA125 did not improve sensitivity for preclinical diagnosis [59]. In addition to this study, five other panels were evaluated, none of which improved upon results with CA125 alone. Zhang *et al.* created a large scale database of 1129 proteins, combining information from ovarian cancer tissues and normal tissues, to enable tumor marker discovery [60]. In 2007, Polanski and Anderson found that out of the 1261 proteins believed to be differentially expressed in human cancer, only nine have been approved as “tumor associated antigens” by the Food and Drug Administration (FDA) [61]. Despite the wide abundance of reported candidate-biomarkers in the literature, only a few proteins are then verified and validated for clinical use. The reasons of this lack of correspondence between laboratory data and clinical setting lies in the inability of current technologies to significantly verify the presence of the promising biomarkers in patient samples and the failure to identify proteins with high specificity for a particular disease.

In 2009, the FDA cleared the OVA1 test to assess preoperatively the risk of ovarian cancer among women with known pelvic masses. The test represents a useful tool to assist physicians in determining which patient would benefit from referral to a specialist surgeon, who would be able to perform debulking and staging surgeries that form the basis of optimal care for ovarian cancer. The OVA1 test is an *in Vitro* Diagnostic Multivariate Index Assay (IVDMIA) of the following five proteomic biomarkers: CA125, transthyretin (prealbumin), apolipoprotein A1, beta 2 microglobulin and transferrin. These biomarkers, other than CA125, were part of seven biomarkers measured by SELDI MS analysis in serum specimens from multiple centers [62,63]. Unfortunately, the reproducibility of the SELDI assay was not adequate for the routine clinical use; thus, only four proteomic biomarkers measured by immunoassay in addition to CA 125 were selected for the validation of the immunoassay-based OVA1 test. Proteomic profiling by mass spectrometry may not be adequate as diagnostic tool, but the profiles that differentiate tumoral tissue from normal tissue may help to identify the proteins candidate as potential biomarkers [64,65].

To conclude, critical assessment of the results has shown significant shortcomings and uncertainties in regard to the reproducibility of the findings and identity of the proteins behind the pattern peaks; thus, the validation of the newly discovered biomarkers still remains the most challenging aspect of clinical proteomics, whereas utilizing pre-diagnostic samples for discovery may be helpful in developing validated early detection panels [66].

## 7. Proteomic Profiling for Targeted Therapies

Proteomic technologies may help to analyze protein signaling pathways to define the preferred targets of patient-tailored molecular therapy. Assessing quantity and functionality of proteins, taking into account the genetic, epigenetic and proteomic features of ovarian cancer cells, may guide treatment decisions. Target therapies may be effective for the subset of patient who harbor tumors with specific protein network defects; thus, the proteomic approach may help to select patients who truly benefit from select molecular therapy.

Posadas *et al.* examined epidermal growth factor receptor (EGFR) and downstream pathways in a phase II clinical trial of gefitinib in ovarian cancer. Notably, the author observed a lack of association between biochemical and clinical effects, since a decrease in the quantity of both total and phosphorylated EGFR in tumor tissue with the administration of gefitinib was not associated with a clinical benefit [67]. Even c-KIT and the platelet-derived growth factor receptor (PDGFR) have been studied as potential molecular target in ovarian cancer [68], and several other trials with proteomic endpoints are still ongoing [4].

Maloney *et al.* found protein expression changes in ovarian cancer cells in response to HSP90 inhibitor 17AAG [69]. The histone deacetylase inhibitor, thiol-specific antioxidant (TSA), was shown to increase acetylation by 30% above control levels. Of note, when 17AAG was added together with TSA to ovarian cancer cells, a significant decrease in acetylation was observed compared with both the untreated control and TSA alone-treated cells. Combination studies of TSA with 17AAG gave a combination index of 2.92 ± 0.64 (SD, *n* = 3). These results showed that acetylation levels are altered following HSP90 inhibition, suggesting that effects on protein acetylation may potentially play a role in the mechanism of action of HSP90 inhibitors. They also suggested that care should be taken in combining histone deacetylase and HSP90 inhibitors to decrease cell proliferation.

Genetic aberrations in *BRCA 1/2* genes are the best validated genetic molecular target in ovarian cancer. The poly (ADP-ribose) polymerase (PARP) inhibitors represent a considerable promise for the treatment of cancers in *BRCA 1/2* mutation carriers. This therapeutic approach exploits a synthetic lethal strategy to target the specific DNA repair pathway in these tumors [70]. Particularly, in recent clinical trials, *BRCA* mutations carriers showed favorable responses to the PARP inhibitor olaparib compared with patients without *BRCA* mutations [71]. Interestingly, Stefansson *et al.* showed that the development of a subset of sporadic tumors is similar in genomic alterations to that of *BRCA1/2*-related tumors. The importance of these observations lies in the potential benefit from targeted therapy, through the use of PARP inhibitors, for a much larger group of patients than the *BRCA1/2* germline mutation carriers [72].

However, despite the good premises, this wealth of information has yielded little in changing the clinical care from a treatment perspective [4].

## 8. Conclusions

Ovarian cancer represents one of the four main gynecological tumors with an increasing incidence and the poorest survival of all gynecological cancer sites. In the last few decades, new approaches at the molecular and cellular level have given useful insights to improve early detection and to personalize treatment strategies. Ovarian cancer represents a heterogeneous disorder with different epidemiology, precursor lesions, gene expression profiles, biomarkers, therapy responses and prognosis, according to each distinct histotype. In order to identify a gene-proteomic signature that may bring to the definition of specific profiles, gene and protein expression analyses have been widely introduced. Particularly, the development and implementation of advanced investigation technologies lead to a substantial change of research objectives toward a genetic and proteomic setting.

Proteomic techniques characterize all proteins in a biological system. One of the main goals of proteomics is the identification of biomarkers for diseases from tissues and body fluids. The advancing techniques for proteomics have shown promise in a variety of studies identifying several potential candidate biomarkers and prognostic indicators of outcome and response to therapy, but few have turned out to be useful in the clinical setting. The use of a combination of biomarkers may improve specificity and sensitivity for early detection of ovarian cancer. Thus, in the last few years, several multiplex assays integrating different candidate biomarkers, sometimes associated with radiological and clinical features, have been developed (such as ROMA). However, a number of questions about the current paradigm for biomarker development in the diagnostic setting still remain open, and in future research, utilizing pre-diagnostic samples for discovery may be necessary to develop validated early detection panels. Despite the wide abundance of candidate-biomarkers, only a few proteins have been validated for clinical use by the FDA. Among these, HE4 has been approved to monitor recurrence or progression of epithelial ovarian cancer, and the OVA-1 test has been cleared to assess preoperatively the risk of ovarian cancer among women with known pelvic masses. At present, the development of an effective strategy for early detection of ovarian cancer is still a work in progress. On the other hand, proteomics have given useful insight into the perturbations of signaling pathways within tumor cells, allowing for the discovery of new drug targets and helping to select patients who truly benefit from select molecular therapy. However, this wealth of information has yielded little in changing clinical care from a treatment perspective.

In conclusion, parallel to the increasing optimism for proteomic expression profiling, an increasing need for accurate and rigorous research is required to answer clinically relevant questions, since, to date, integrating the biochemical information into clinical management still remains an unresolved challenge.

## Figures and Tables

**Table 1:** Wide genetic panel involved in ovarian cancer pathogenesis.

Tumor suppressor genes	Oncogenes	Imprinted tumor suppressor genes
*ARHI*, *RASSF1A*, *DLEC1*, *SPARC*,	*RAB25*, *EVI1*, *EIF5A2*, *PRKCI*,	*ARHI*, *PLAGL1*, *PEG3*
*DAB2*, *PLAG1*, *RPS6KA2*, *PTEN*,	*PIK3CA*, *MYC*, *EGFR*, *NOTCH3*,	
*OPCML*, *BRCA2*, *ARL11*, *WWOX*,	*KRAS*, *ERBB2*, *PIK3R1*, *CCNE1*,	
*TP53*, *DPH1*, *BRCA1*, *PEG3*	*AKT2*, *AURKA*	

**Table 2:** Promising biomarkers discovered by proteomic technology for ovarian cancer diagnosis.

Authors	Identified biomarkers	Regulation in cancer
Cortesi *et al.* (2011) [42]	Annexin-5 (ANXA5)	↓
	Phosphatidylethanolamine-binding protein 1 (PEBP)	↓
	Glutathione *S*-transferase A2 (GSTA2)	↓
	Galectin-3 (LEG3)	↓
	Protein S100-A8-calgranulin A (S100A8)	↑
	Retinol binding protein (RET1)	↓
Petri *et al.* (2009) [43]	Fibrinogen alpha fragment	↑
	Collagen alpha 1 (III) fragment	↑
	Fibrinogen beta NT fragment	↑
Li *et al.* (2009) [44]	Pyridoxine II	↓
	Pyridoxine-III	↑
	Heat shock protein 27 (HSP27)	↑
	Heat shock protein 60 (HSP60)	↑
	Mitochondrial short-chain enoyl-CoA hydratase	↑
	Prohibitin	↑
Jackson *et al.* (2007) [45]	Vitamin E-binding plasma protein, Afamin	↓
An *et al.* (2006) [46]	Annexin-1 (ANXA1)	↑
	NM23-H1	↑
	Protein phosphatase-1	↑
	Ferritin light chain	↑
	Proteasome alpha-6	↑
	*N*-acetyl glucosamine kinase (NAGK)	↑

**Table 3:** Modification in protein expression in tissue and interstitial fluid.

Protein	FOLD CHANGE * tumoral *vs.* normal tissue	*p*-value	FOLD CHANGE * TIF *vs.* NIF	*p*-value
ANXA5	−1.88 ± 0.48	<0.0001	−5.605 ± 3.29	<0.01
PEBP	−4.21 ± 2.90	<0.01	−2.82 ± 0.69	<0.0001
GSTA2	−4.67 ± 1.88	<0.0001	−27.39 ± 21.24	<0.01
LEG3	−2.19 ± 0.69	<0.0001	−5.10 ± 4.42	<0.05
S100A8	3.67 ± 1.50	<0.01	3.58 ± 1.11	<0.0001
RET1	−6.33 ± 3.30	<0.001	−5.01 ± 4.28	<0.05

* The fold change indicates the direction and the magnitude of the change in expression level. Data are expressed as the mean ± standard deviation.
